# Psychopathological features, neuroendocrine correlates and clinical chemistry markers in bariatric surgery candidates: preliminary assessments

**DOI:** 10.1192/j.eurpsy.2025.2026

**Published:** 2025-08-26

**Authors:** E. Diadema, M. Simoncini, M. Violi, A. Bordacchini, G. Giannaccini, L. Betti, L. Palego, A. Cimino, S. Fantasia, M. Nannipieri, C. Carmassi

**Affiliations:** 1 University of Pisa, Pisa; 2 University of Siena, Siena, Italy

## Abstract

**Introduction:**

Obesity represents a heterogeneous group of clinical conditions, underpinned by a multifactorial pathogenesis. People affected by severe obesity could be eligible for Bariatric Surgery (BS). Generally, researchers agree on the complex interplay between a variety of biochemical and neuroendocrine factors in determining body weight regulation, as well as on the quite common co-exhibition of severe obesity and psychopathological symptoms. Both obesity and mood disorders resulted as chronic low grade pro-inflammatory states and it has been stressed the relevance of traumatic life events in overweight conditions, but few is known about underlining trajectories and neurobiological correlates. BS candidates have high rates of lifetime psychiatric disorders, supporting a comprehensive assessment of psychopathological and peripheral biomarkers in this population.

**Objectives:**

Aim of this cross-sectional survey was the investigation of possible relationships between hematochemical parameters and specific psychopathological features in a sample of BS candidates.

**Methods:**

Seventy-seven subjects with severe obesity undergoing the BS preoperative multidisciplinary evaluation at the University Hospital of Pisa were investigated. Psychopathological data were obtained by self-report instruments exploring a series of full-blown and sub-threshold symptoms of mood and post-traumatic-stress disorders, as well as for emotional eating features: the *Mood spectrum-self report (MOODS-SR) lifetime*; the *Trauma and Loss Spectrum self-report (TALS-SR) lifetime*; the *Emotional Eating Scale (EES).* As concerns the biochemical assessment, we considered morning cortisol plasma levels and blood cell counts. Non-parametric Spearman correlations were applied. The statistical threshold was set up at P ≤ .05.

**Results:**

We found significant negative correlations between cortisol plasma levels measured in the morning and sleep (P=.001) or appetite disturbances (P=.04), as well as total altered mood scores (P=.001). Significant positive correlations emerged between Platelet count and total depression scores (P=.042), appetite disturbances (P=.027), TALS-SR domain 3 score (P=.0069), as well as the anger (P=.006), the anxiety (P=.025) and the total components (P=.015) in the EES. Interestingly, there were significant positive correlations also between Platelets-to-Lymphocytes Ratio and the domains 3 (P=.015) and 4 (P=.025) of TALS-SR questionnaire.

**Conclusions:**

These preliminary correlations suggest that in severe obesity (or, almost, in a subgroup of patients), post-traumatic stress features, mood, sleep and appetite disturbances could be related to a lower basal hypothalamic-pituitary-adrenal axis activity and higher inflammatory parameters, especially those linked to platelet status.

**Disclosure of Interest:**

None Declared

